# Lamin B1 is a potential therapeutic target and prognostic biomarker for hepatocellular carcinoma

**DOI:** 10.1080/21655979.2022.2057896

**Published:** 2022-04-18

**Authors:** Yongyu Yang, Lei Gao, Junzhang Chen, Wang Xiao, Ruoqi Liu, Heping Kan

**Affiliations:** a Department of Hepatobiliary Surgery, Nanfang Hospital, Southern Medical University, Guangzhou, Guangdong, China; b Department of Neurosurgery, Nanfang Hospital, Southern Medical University, Guangzhou, Guangdong, China

**Keywords:** Hepatocellular carcinoma, LMNB1, therapeutic target, prognostic biomarker, nomogram

## Abstract

Hepatocellular carcinoma (HCC) is an aggressive malignancy. Previous studies have found that lamin B1 (LMNB1) contributes to the development of human cancers. However, the biological functions and prognostic values of LMNB1 in HCC have not been adequately elucidated. In our present research, the expression pattern of LMNB1 was analyzed. The prognostic values of LMNB1 were evaluated by Kaplan–Meier survival analysis and Cox proportional hazards regression analysis. The effects of LMNB1 on HCC progression were assessed by Cell Counting Kit-8 (CCK-8), colony formation, wound healing, Transwell and in vivo xenograft assays. The mechanisms of LMNB1 in HCC progression were elucidated by gene set enrichment analysis (GSEA) and loss-of-function assays. Besides, a nomogram for predicting overall survival (OS) was constructed. The results demonstrated that LMNB1 was overexpressed in HCC and that increased LMNB1 expression predicted a dismal prognosis. Further experiments showed that LMNB1 facilitated cell proliferation and metastasis in HCC. Functional enrichment analysis revealed that LMNB1 modulated metastasis-associated biological functions such as focal adhesion, extracellular matrix, cell junctions and cell adhesion. Mechanistically, we revealed that LMNB1 promoted HCC progression by regulating the phosphatidylinositol 3-kinase (PI3K) and mitogen-activated protein kinase (MAPK) pathways. Moreover, incorporating LMNB1, Ki67 and Barcelona Clinic Liver Cancer (BCLC) stage into a nomogram showed better predictive accuracy than the Tumor-Node-Metastasis (TNM) stage and BCLC stage. In conclusion, LMNB1 may serve as an effective therapeutic target as well as a reliable prognostic biomarker for HCC.

## graphical abstract

**Figure:**
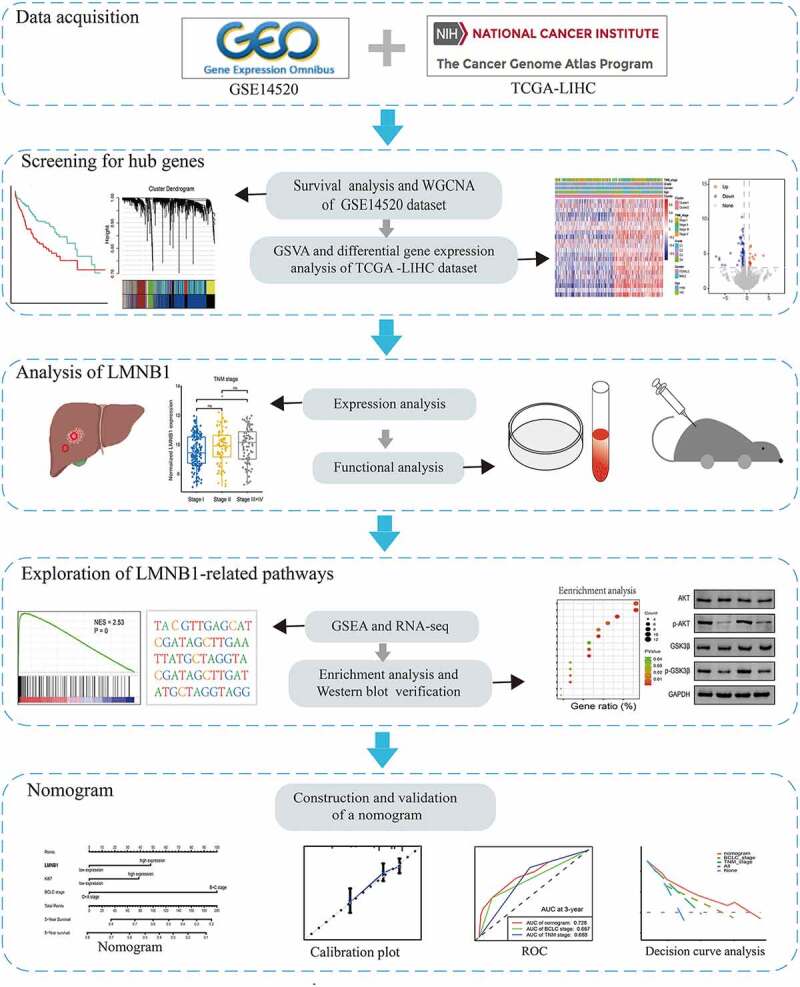

## Introduction

Hepatocellular carcinoma (HCC) is the sixth most common malignancy in the world and is the third-highest cause of cancer-related death [1]. Although several therapeutic strategies, such as hepatectomy, transarterial chemoembolization (TACE), radiofrequency ablation and immunotherapy, have been established [2,3], the clinical outcome of HCC is still unsatisfactory. The high mortality of HCC is caused by its high metastasis and recurrence rates [4]. Patients with HCC are often diagnosed at an advanced stage [5]. Targeted therapy is one of the most important treatments for patients with advanced HCC [6]. Unfortunately, Tang et al. reported that only approximately 30% of patients with HCC benefit from targeted therapy [7]. Therefore, there is an urgent need to identify effective therapeutic targets by exploring the underlying molecular changes in HCC.

LMNB1 belongs to the type V intermediate filament protein family and is found below the inner nuclear membrane [8,9]. LMNB1 has functional roles in transcription, cell cycle progression, nuclear stability, DNA replication and repair, cell proliferation and differentiation [10,11]. To date, numerous studies have reported that LMNB1 is dysregulated in different cancers, including colorectal cancer, clear-cell renal carcinoma, pancreatic cancer and gastric cancer [12–15]. LMNB1 promotes tumor progression through multiple mechanisms. For example, overexpression of LMNB1 in pancreatic cancer cells greatly enhances anchorage-independent growth and migration abilities by targeting Sp1 transcription factor (Sp1) [14]. Overexpression of LMNB1 promotes colon cancer progression by inducing mitotic catastrophe [12]. Previous studies found that LMNB1 was significantly upregulated in HCC and present in patient plasma, thus LMNB1 may be a potential diagnostic biomarker for detecting early-stage HCC patients [16,17]. However, the prognostic values, biological functions and underlying mechanisms of LMNB1 in HCC have yet to be elucidated.

The TNM stage and BCLC stage are routinely used for HCC treatment and prognostication [18,19]. However, different clinical outcomes have been observed in HCC with the same TNM or BCLC stage [20,21]. These findings indicate that the current HCC staging systems fail to provide complete prognostic information. One of the reasons may be that the current staging systems ignore the complex molecular mechanisms of HCC. Molecular biomarkers may provide valuable information in the prognostic prediction of HCC. Zhou et al. found that Absent in melanoma 1-like (AIM1L) could predict OS for HCC patients [22]. Recent studies have combined several molecular biomarkers to create predictive signatures [23,24]. However, the roles of these markers lack experimental validation, and the predictive signatures are not utilized in clinical practice. Thus, it is imperative to explore a more accurate predictive nomogram to guide precise individual treatment for HCC patients.

In this study, we hypothesized that LMNB1 serves as an effective therapeutic target as well as a prognostic biomarker for HCC. To validate this hypothesis, we conducted a combined analysis of public databases and clinical specimens to explore the expression pattern and clinical significance of LMNB1 in HCC. We then investigated the biological functions of LMNB1 in HCC by a series of in vitro and in vivo assays. Moreover, we provided insight into LMNB1-related pathways through GSEA, RNA sequencing (RNA-seq) and pathway enrichment analysis. Finally, we constructed a nomogram to predict the OS of HCC patients. We hope this study will contribute to our understanding of targeted therapy and prognosis evaluation of HCC.

## Materials and methods

### Data collection

High-throughput transcriptome sequencing mRNA expression data and clinical parameters of the liver hepatocellular carcinoma (LIHC) dataset were downloaded from The Cancer Genome Atlas (TCGA). The TCGA-LIHC dataset contains information on 371 tumor samples. The histological types of the LIHC dataset included HCC, clear cell adenocarcinoma, combined hepatocellular carcinoma and cholangiocarcinoma. The normalized microarray GSE14520 dataset and its corresponding clinical parameters were obtained from the Gene Expression Omnibus (GEO). The GSE14520 dataset contains 247 HCC samples. Samples without complete clinical parameters and samples whose histological type was not HCC were removed. Finally, 364 patients from the TCGA-LIHC dataset and 220 patients from the GSE14520 dataset were used for subsequent analysis.

### Screening prognostic genes in the GSE14520 dataset

In the GSE14520 dataset, the median mRNA expression value was selected as a cutoff value. HCC patients were divided into high mRNA expression and low mRNA expression two groups according to the cutoff value. Then, we performed Kaplan–Meier analysis of all the mRNA expression profiles to screen out the prognostic genes by using the ‘survminer’ R package. P < 0.05 was selected as a cutoff value to screen prognostic genes.

### Weighted gene co-expression network analysis (WGCNA)

WGCNA is widely used to unravel the correlation between gene modules and clinical phenotypes [25]. We conducted WGCNA based on the prognostic genes through the R package ‘WGCNA’. First, the correlations between the prognostic genes were calculated by Pearson’s method to establish an adjacency matrix. Then, an optimal soft threshold was chosen through the scale-free topological fit test. R^2^ > 0.9 was selected as a scale-free fit to obtain a high-confidence scale-free network. Second, according to the optimal soft threshold, a topological overlap matrix (TOM) was established based on the adjacency matrix. Subsequently, by using a dynamic tree cut algorithm, hierarchical clustering was conducted for module screening. The cutreeDynamic function of the WGCNA package was used for tree pruning of the hierarchical clustering dendrograms. Correlations between the gene modules and clinical phenotypes were further calculated to screen gene modules that were significantly relevant to the metastasis and survival status of HCC.

### Gene set variation analysis (GSVA) and identification of metastasis score-based subgroups

The normalized mRNA expression data of the TCGA-LIHC dataset were used for GSVA [26]. We obtained metastasis-associated gene expression signatures from the C2 curated gene sets in the Molecular Signatures Database (https://www.gsea-msigdb.org/gsea/msigdb). We calculated the metastasis scores for HCC patients based on the 22 selected metastasis-associated gene sets (Supplementary Table 1) through the R package ‘GSVA’. To discover metastasis score-based subgroups, we performed unsupervised clustering based on the metastasis score profiles by using the ‘ConsensusClusterPlus’ R package. The maximum K value of 9 with 80% item resampling and 50 resamplings was used for clustering. The optimal K value of clusters was 2 according to the cumulative distribution functions (CDFs). Thus, HCC patients were classified into cluster 1 and cluster 2 two groups.

### Screening of differentially expressed genes (DEGs)

To discover metastasis-associated hub genes in HCC. We used the ‘edgeR’ package to identify DEGs between cluster 2 (high metastasis score) and cluster 1 (low metastasis score) patients. Genes with false discovery rate (FDR) < 0.01 and log2-fold-change ≥ 1 were considered significant DEGs that were upregulated in HCC with high metastasis scores.

### Cell lines, culture conditions and clinical specimens

The normal hepatic cell line LO2 and HCC cell lines MHCC-97H, Huh7, Hep3B and HepG2 were purchased from the Chinese Academy of Sciences (Shanghai, China). These cells were cultured in DMEM (Gibco) supplemented with 10% FBS (Gibco). All cells were maintained in a humidified incubator with 5% CO2 at 37°C.

Tumor and paired peritumoral specimens were obtained from 20 HCC patients who underwent partial hepatectomy at Nanfang Hospital of Southern Medical University. Before surgery, none of the patients received specialized treatment, such as targeted therapy, immunotherapy or TACE. Patient specific information could be found in Supplementary Table 2. All patients voluntarily signed informed consent before the study began. This study was approved by the Ethics Committee of Nanfang Hospital of Southern Medical University.

### Lentiviral construction and cell transfection

Lentiviral small hairpin RNA targeting LMNB1 (lv-sh-LMNB1) and empty lentiviral vector (lv-sh-con) were purchased from Hanbio (Shanghai, China). The sequence of sh-LMNB1 was 5’- GATCCGCGCTTGAAGAACACTTCTGAATTCAAGAGATTCAGAAGTGTTCTTCAAGCGTTTTTTG -3’. HepG2 and Hep3B cells were infected with lentivirus to construct stably transfected cell lines. After 72 h, we removed negative cells through puromycin selection (Solarbio).

### Immunohistochemistry (IHC)

Immunohistochemistry was conducted as previously reported [27]. Antibodies against LMNB1 (1:200, Santa Cruz, sc-377,000), matrix metallopeptidase 11 (MMP11) (1:100, Abcam, ab119284) and Ki67 (1:200, Proteintech, 27309-1-AP) were used for IHC. Image-Pro Plus 6.0 software (IPP6) was used to analyze the IHC results. The integrated optical density in each microscopic view (IOD/area) was measured to quantify the staining intensity.

### CCK-8 assay

Stable HCC cell lines with or without LMNB1 knockdown were seeded into 96-well plates. The cell density was set as 200 cells per well. After incubation for 24 h, ten microliters of CCK-8 solution (Solarbio, CA1210) were added to the cells in each well. The optical density at 450 nm was calculated by a microplate reader.

### Colony formation assay

Stable HCC cell lines with or without LMNB1 knockdown were seeded into 6-well plates. The cell density was set as 400 cells per well. After incubation for 14 days, the medium was removed, and the cells were washed three times with PBS. Then, the cells were fixed with 4% paraformaldehyde and stained with 0.1% crystal violet solution. The colony numbers were counted to evaluate cell proliferation abilities among different groups.

### Wound healing assay

LMNB1 knockdown or control HepG2 and Hep3B cells were cultured in 6-well plates. After the cell density reached 90%, we made a wound in each well plate using a 100 μl pipette tip. Then, the medium was replaced with serum-free DMEM. We recorded the wound healing area at 0 h, 24 h and 48 h to evaluate the cell migration ability.

### Transwell invasion assay

Transwell invasion assays were conducted with Matrigel-coated chambers (8-μm pore size; 24-well; Corning). A total of 5 × 10^4^ cells with or without LMNB1 knockdown in DMEM without FBS were seeded in the upper chamber. DMEM with 20% FBS was added to the lower chamber. The cells that had traversed the membrane were fixed with 4% paraformaldehyde and then stained with 0.1% crystal violet. Finally, the invading cells were observed under a microscope.

### Western blot analysis

Western blot analysis was performed following our previous description [28]. Antibodies against LMNB1 (1: 1000, Santa Cruz, sc-377000), Snail (1: 1000, CST, # 3879), MMP11 (1: 1000, Abcam, ab119284), Slug (1: 1000, CST, # 9585), N-cadherin (1: 1000, CST, # 13116), Akt (1: 1000, CST, # 4691), GSK-3β (1: 2000, Abcam, ab93926), Phospho-Akt (p-Akt) (1: 2000, CST, #4060), Phospho-GSK-3β (p-GSK-3β) (1: 1000, Abcam, ab75745) and glyceraldehyde-3-phosphate dehydrogenase (GAPDH) (1: 10000, Abcam, ab8245) were used for this assay. GAPDH was selected as the internal control. Finally, an electrochemiluminescence imaging analysis system was used to detect the signals of the bands.

### In vivo xenograft assay

We purchased BALB/c male nude mice from the Animal Center of Southern Medical University. All nude mice were 4–6 weeks old. This animal experiment was approved by the Southern Medical University Experimental Animal Ethics Committee. Each nude mouse was subcutaneously inoculated with 5.0 × 10^6^ HepG2 cells that were infected with lv-sh-LMNB1 vector or lv-sh-con vector. Tumor size was measured weekly. The mice were sacrificed in the fourth week. Tumor volume (mm^3^) was calculated with the equation: V = L× W^2^ × 0.5.

### RNA sequencing (RNA-seq), functional annotation, and pathway enrichment analysis

Total RNA derived from HepG2 cells infected with lentivirus was quantified and then used for RNA-seq. DEGs were screened between the lv-sh-LMNB1 and lv-sh-con groups by the ‘edgeR’ package. The DEGs with fold change > 1.5 and p < 0.05 were used for subsequent analysis. We further performed Gene Ontology (GO) and Kyoto Encyclopedia of Genes and Genomes (KEGG) pathway enrichment analyses on the DEGs. The enrichment analysis was performed on the Database for Annotation, Visualization, and Integrated Discovery (DAVID, Version 6.8; https://david.ncifcrf.gov/). According to the median LMNB1 expression value, HCC patients were labeled LMNB1^high^ or LMNB1^low^. Then GSEA [29] was performed to identify the significant pathways enriched by LMNB1. The gene set file used was ‘c2. all. v7.1. symbols. gmt’.

### Cox proportional hazards regression analysis

To determine the risk factors for HCC, we performed univariate Cox analysis among LMNB1, Ki67, age, gender, alanine aminotransferase (ALT), alpha fetoprotein (AFP) and BCLC stage from the GSE14520 dataset. Variables with P < 0.05 were considered as risk factors for overall survival (OS). All statistically significant risk factors identified by the univariate Cox analysis were subsequently included in the multivariate Cox analysis. The hazard ratio and 95% confidence interval of each variable were also calculated.

### Construction and validation of a nomogram

Schönfeld test was used to verify the proportional hazard (PH) assumption. The independent risk factors that meet the PH assumption were used to constructed a predictive nomogram. Then, we drew calibration curves and receiver operating characteristic (ROC) curves to evaluate the performance of the nomogram. Moreover, we performed decision curve analysis (DCA) to systematically compare the clinical application among the nomogram, TNM stage and BCLC stage.

### Statistical analysis

Statistical analysis was performed by SPSS software 22.0 (IBM Corp, New York, USA), R software (version 4.1.2), and Prism 8.0 (GraphPad Software, San Diego, CA, USA). Statistical differences between two independent groups were evaluated by Student’s t test. Cox proportional hazards regression model and Kaplan–Meier survival analysis were used to assess the prognostic value of LMNB1. Statistical significance was set at P < 0.05.

## Results

The present study aimed to explore the clinical significance, biological functions and underlying mechanisms of LMNB1 in HCC by bioinformatic analysis and fundamental experiments. Our analysis revealed that LMNB1 was upregulated in HCC and that increased LMNB1 expression was positively correlated with advanced TNM stage, worse histological grade, high risk of tumor metastasis and dismal prognosis. Functional and mechanistic studies revealed that LMNB1 promotes HCC proliferation and metastasis and regulates the PI3K/MAPK signaling pathway. Another purpose of our study was to construct a nomogram to predict the OS of HCC patients. We incorporated LMNB1, Ki67 and BCLC stage into a nomogram. Surprisingly, the performance of this nomogram was better than that of the TNM stage or BCLC stage.

### LMNB1 is associated with HCC metastasis and is overexpressed in HCC

**Figure 1.**
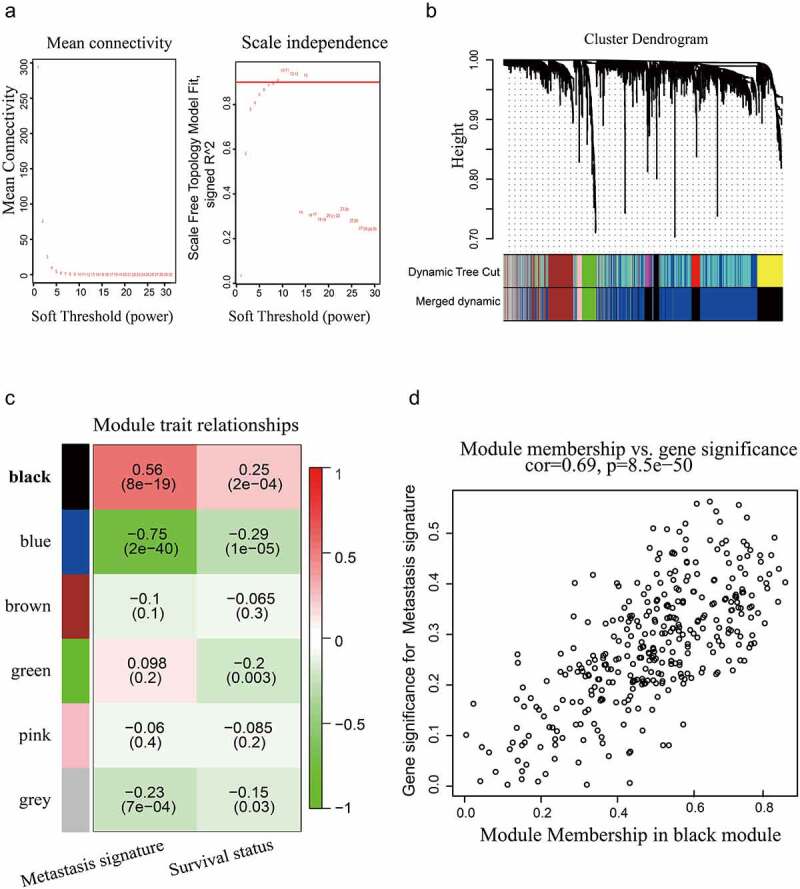
WGCNA based on the GSE14520 dataset. (a) Optimal soft-thresholding power was selected according to the network topology analysis. (b) Five gene modules were identified in the clustering dendrogram of the prognostic genes. (c) Correlations between the gene modules and clinical traits. (d) The correlation between the black gene module and HCC metastasis is shown in the scatter plot.

**Figure 2.**
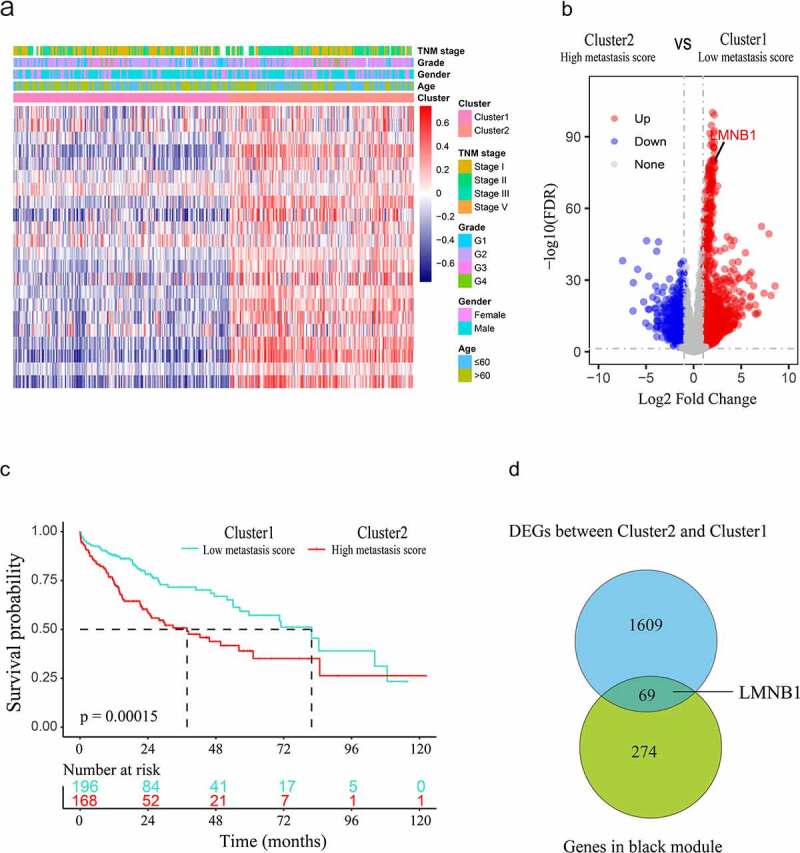
Screening hub genes from the black module and DEGs. (a) Heatmap of the clusters defined by metastasis-associated gene sets based on consensus clustering. (b) DEGs between cluster 2 (high metastasis score) and cluster 1 (low metastasis score) are shown in the volcano plot. (c) Survival curve of HCC patients in cluster 1 and cluster 2. (d) The hub genes of HCC are shown in the Venn diagram.

We performed survival analysis of mRNA expression profiles and identified 1799 prognostic genes from the GSE14520 dataset. The above prognostic genes were subjected to WGCNA, thus leading to the discovery of pivotal mRNAs that modulate HCC metastasis. We selected 0.9 as a scale-free topology fit index and 6 as a soft threshold power to generate the TOM (Figure 1(a)). After applying the dynamic tree cut algorithm, five gene modules were identified among all prognostic genes (Figure 1(b)). The gray module that had no significance was removed. We estimated the association between modules and clinical traits. As shown in the heat map, the correlation between the black module and HCC metastasis was strongest (R = 0.56, P < 0.001, Figure 1(c)). Then, the correlation between module membership (MM) and gene significance (GS) was calculated by Pearson correlation analysis. The scatter plot of MM vs. GS revealed that the black module was positively correlated with HCC metastasis (Figure 1(d)). The black module contained 343 metastasis-associated genes (Supplementary Table 3). In the TCGA-LIHC dataset, we further characterized HCC patients with metastasis scores by using GSVA algorithm based on metastasis-associated gene sets. Then, 364 HCC samples with metastasis scores were used for unsupervised clustering. Hierarchical clustering revealed two main subgroups (cluster 1 and cluster 2, Figure 2(a)). Cluster 1 was assigned to the low metastasis score subgroup, and cluster 2 was assigned to the high metastasis score subgroup. Differential expression analysis revealed that 1678 DEGs were upregulated in cluster 2 (Figure 2(b)), (Supplementary Table 4). The two subgroups showed distinct survival outcomes. HCC patients in cluster 1 had a significantly longer OS rate than those in cluster 2 (Figure 2(c)). As the Venn diagram (Figure 2(d)) shows, 69 overlapping genes (Supplementary Table 5) were identified as oncogenes of HCC. LMNB1 was one of the oncogenes that was significantly associated with HCC metastasis, indicating that LMNB1 may promote HCC progression by inducing cell metastasis.

**Figure 3.**
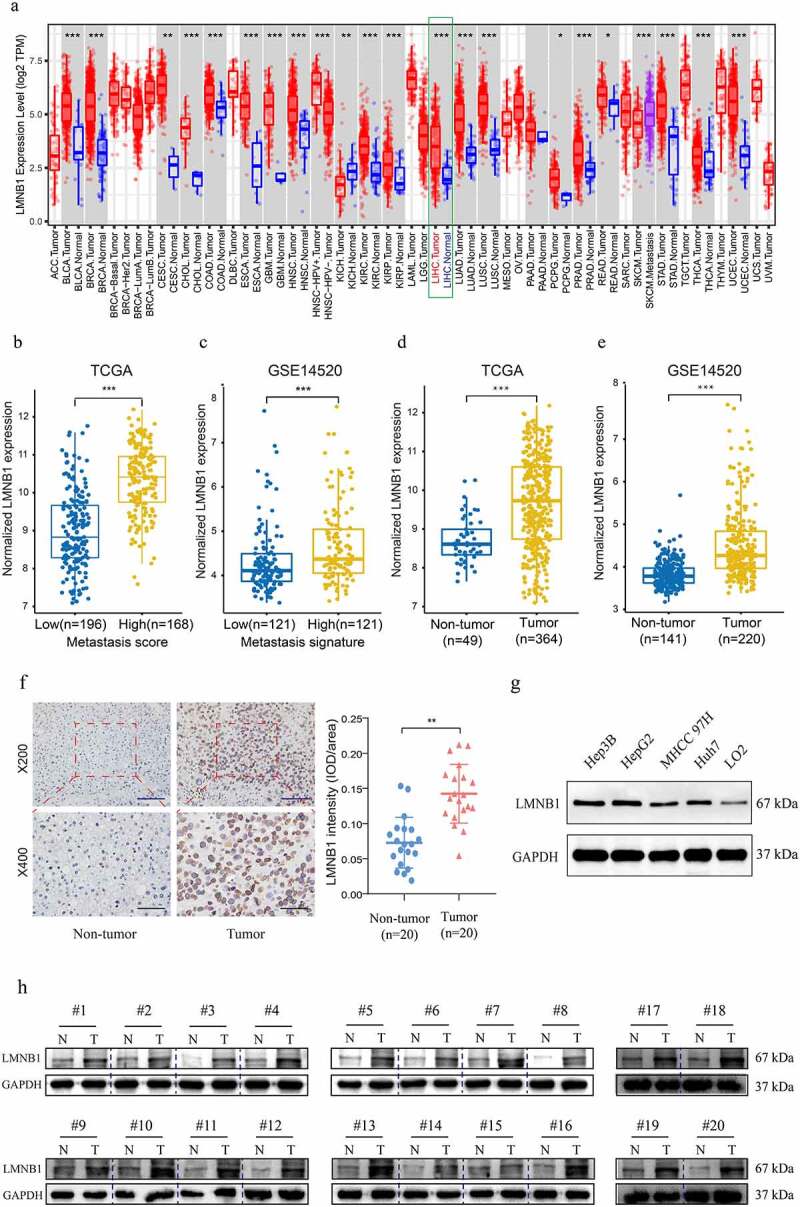
Expression analysis of LMNB1 in HCC. (a) LMNB1 was abnormally expressed in different malignancies in the TIMER2 database. (b) LMNB1 was upregulated in patients with high metastasis scores. (c) LMNB1 was upregulated in patients with high metastasis signatures. (d, e) In the GSE14520 dataset (d) and TCGA-LIHC dataset (e), LMNB1 was overexpressed in HCC compared to peritumoral tissues. (f) Representative IHC images of HCC tissues and peritumoral tissues. IHC verified that LMNB1 was overexpressed in HCC. Scale bars (up) = 100 μm. Scale bars (low) = 50 μm. (g) Overexpression of LMNB1 was detected in HCC cell lines. (h) Western blot analysis of HCC tissues and peritumoral tissues. *P < 0.05, **P < 0.01, ***P < 0.001.

The expression features of LMNB1 in different types of tumors were evaluated by analyzing transcriptome expression data from the TIMER2.0 database. Compared to normal tissues, LMNB1 was significantly upregulated in most types of tumor tissues (Figure 3(a)). From the TCGA dataset, overexpression of LMNB1 was observed in the high metastasis score patients in comparison with the low metastasis score patients (Figure 3(b)). The same trend could be found between HCC patients with a high metastasis signature and those with a low metastasis signature from the GSE14520 dataset (Figure 3(c)). In addition, gene expression data from both the TCGA and GSE14520 datasets revealed that LMNB1 was significantly upregulated in HCC (Figure 3(d,e)). Furthermore, IHC and western blot analyses results verified that LMNB1 was upregulated in HCC compared to peritumoral tissues (Figure 3(f,h)). The western blot analysis showed a similar result in the LO2 and HCC cell lines (Figure 3(g)).

### Overexpression of LMNB1 is associated with aggressive clinicopathological characteristics

**Figure 4.**
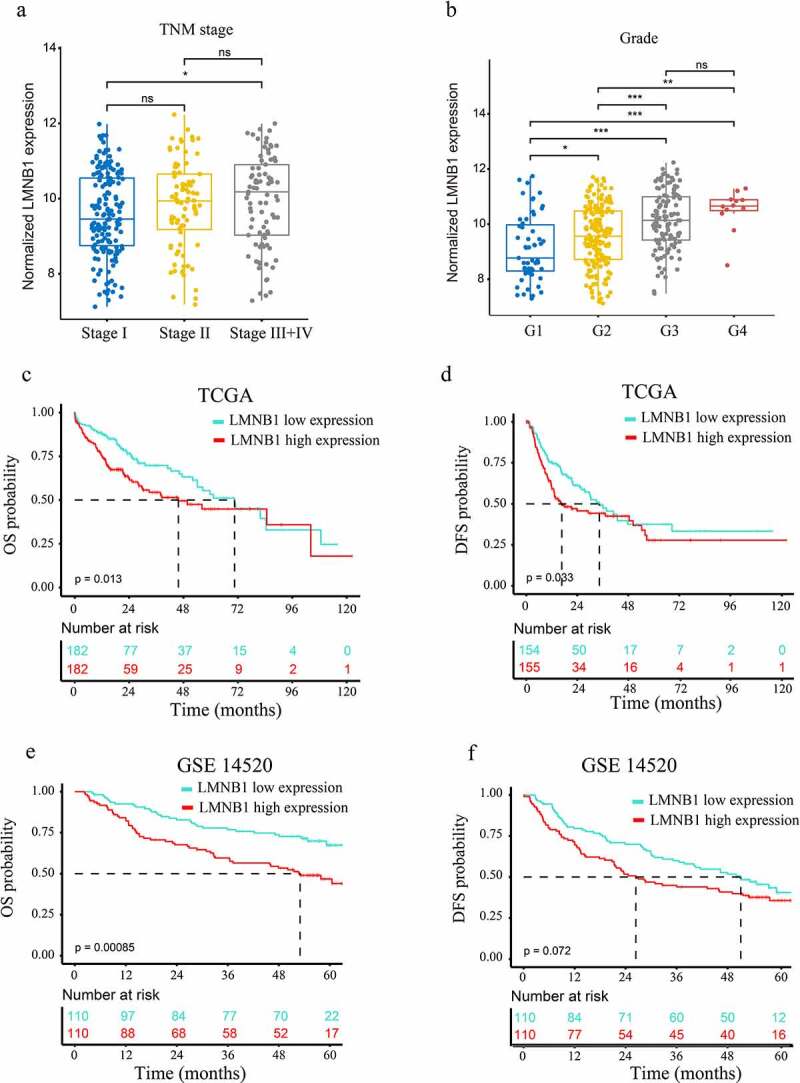
Clinicopathological and prognostic analyses of LMNB1. (a, b) Overexpression of LMNB1 was positively correlated with aggressive clinicopathological features, including advanced TNM stage (a) and worse histological grade (b). (c, d) Survival analyses of OS (c) and DFS (d) between LMNB1^high^ and LMNB1^low^ HCC patients in the TCGA-LIHC dataset. (e, f) Survival analyses of OS (e) and DFS (f) between LMNB1^high^ and LMNB1^low^ HCC patients in the GSE14520 dataset. *P < 0.05, **P < 0.01, ***P < 0.001.

Supplementary Table 6 shows that LMNB1 expression was significantly associated with age (p = 0.007), AFP level (p < 0.001), grade (p < 0.001), tumor status (p = 0.009), metastatic score (p < 0.001) and TNM stage (p = 0.009). No direct effects of gender and BMI were observed on LMNB1 expression (p > 0.05). (Figure 4(a)) show that the expression of LMNB1 in HCC with TNM stage III+IV was significantly higher than those in HCC with TNM stage I. (Figure 4(b)) show that the expression of LMNB1 in HCC with poorer differentiation were significantly higher than those in HCC with good or moderate differentiation.

### Overexpression of LMNB1 indicates a dismal prognosis in patients with HCC

We also evaluated the prognostic significance of LMNB1 in HCC. As shown in the Kaplan–Meier survival curves, overexpression of LMNB1 was correlated with poor OS (P = 0.013, Figure 4(c)) and disease-free survival (DFS, P = 0.033, Figure 4(d)) in the TCGA-LIHC dataset. A similar result was observed for OS in the GSE14520 dataset (P = 0.00085, Figure 4(e)). However, HCC patients with LMNB1^high^ and LMNB1^low^ expression exhibited no significant difference in terms of DFS (P = 0.072, Figure 4(f)). Moreover, univariate Cox analysis revealed that high serum AFP, advanced BCLC stage, overexpression of LMNB1 and overexpression of Ki67 were risk factors for OS. Multivariate analysis revealed that overexpression of LMNB1, overexpression of Ki67 and advanced BCLC stage independently predicted poor OS in HCC (Table 1).

**Table 1.** Univariate and multivariate Cox regression analysis for OS in HCC patients based on GSE14520 dataset (n = 220)

Variables	Univariate analysis			Multivariate analysis		
	HR	95% CI	P value	HR	95% CI	P value
Age (≥ 60 vs < 60)	0.820	0.469–1.433	0.486			
Gender (male vs female)	0.613	0.296–1.269	0.187			
ALT (>50 U/L vs ≤50 U/L)	1.097	0.714–1.685	0.672			
AFP (>300 ng/ml vs ≤300 ng/ml)	1.606	1.049–2.460	0.029	1.257	0.815–1.939	0.301
BCLC stage (B + C vs 0 + A)	3.522	2.264–5.480	<0.001	3.372	2.147–5.294	<0.001
LMNB1 (high expression vs low expression)	2.087	1.341-3.247	0.001	1.805	1.139–2.863	0.012
Ki67 (high expression vs low expression)	1.990	1.284–3.83	0.002	1.614	1.023–2.547	0.040

OS, overall survival. HR, hazard ratio. CI, confidence interval

### Knockdown of LMNB1 inhibits the growth and migration of HCC cells in vitro

**Figure 5.**
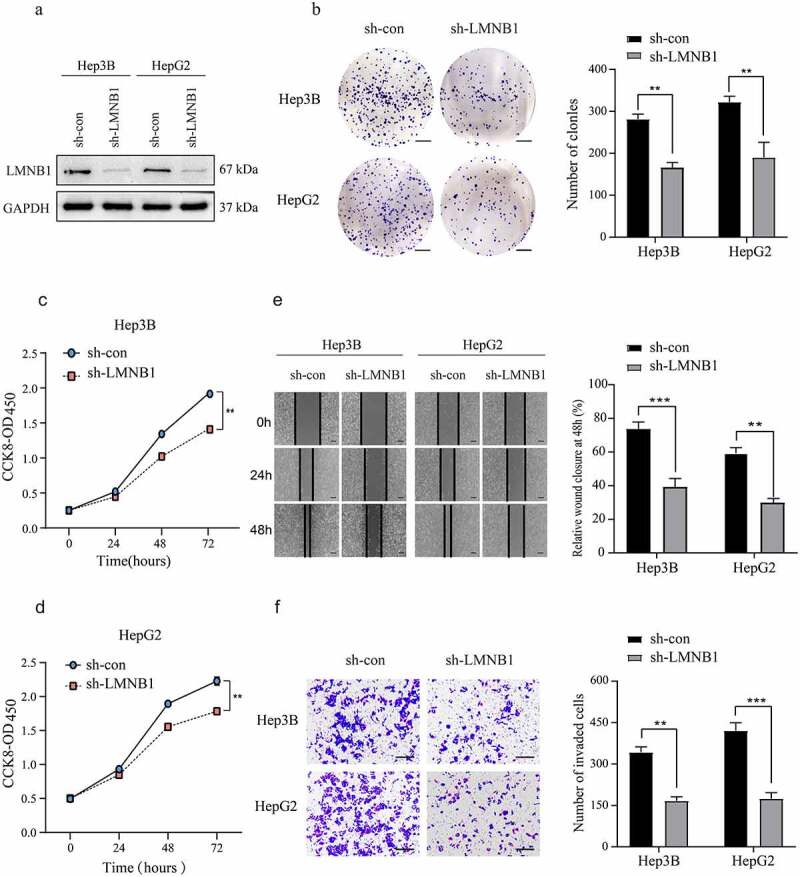
Knockdown of LMNB1 inhibits HCC growth and migration in vitro. (a) LMNB1 was knocked down in Hep3B and HepG2 cells, and the LMNB1 knockdown efficiency was confirmed by western blot analysis. (b) In colony formation assays, LMNB1 knockdown suppressed HCC proliferation. Scale bars = 0.5 cm. (c, d) In CCK-8 assays, LMNB1 knockdown suppressed HCC proliferation. (e) LMNB1 knockdown inhibited HCC migration. Scale bars = 200 μm. (f) LMNB1 knockdown inhibited HCC invasion. Scale bars = 100 μm. *P < 0.05, **P < 0.01, ***P < 0.001.

Bioinformatic analysis revealed that LMNB1 was associated with the metastasis and prognosis of HCC. We stably deleted LMNB1 in HCC cell lines to confirm its tumorigenic roles (Figure 5(a)). Reduced colony formations were observed in LMNB1 knockdown HCC cell lines (Figure 5(b)). In CCK-8 assays, LMNB1 silencing attenuated the proliferation abilities of HCC cell lines (Figure 5(c,d)). In wound healing and Transwell assays, LMNB1 knockdown significantly delayed the wound healing time (Figure 5(e)) and suppressed the invasion of HCC cells (Figure 5(f)). The above findings verified that LMNB1 plays a tumorigenic role in HCC.

### Knockdown of LMNB1 suppresses HCC tumorigenesis in vivo and restrains EMT

**Figure 6.**
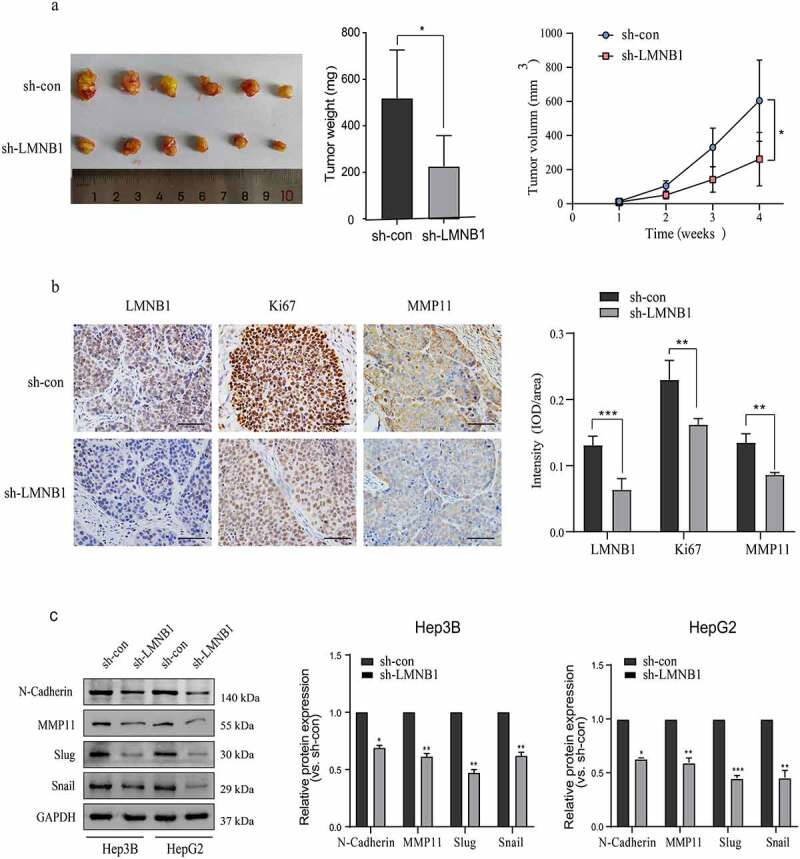
Knockdown of LMNB1 suppresses HCC tumorigenesis in vivo and restrains EMT. (a) The weight and growth curves of the subcutaneous tumors. (b) IHC analysis of LMNB1, Ki67 and MMP11 in subcutaneous tumors. Scale bars = 50 μm. (c) Western blot analysis of EMT-relevant markers, including N-cadherin, Slug, Snail and MMP11. *P < 0.05, **P < 0.01, ***P < 0.001.

To confirm the functional roles of LMNB1 in vivo, a mouse tumorigenesis assay was conducted in our laboratory. The subcutaneous tumors of the lv-sh-LMNB1 group were lighter than those of the lv-sh-con group, and the tumor volumes of the lv-sh-LMNB1 group were relatively smaller than those of the lv-sh-con group (Figure 6(a)). IHC results of the subcutaneous tumors confirmed that LMNB1 had been knocked down in the lv-sh-LMNB1 group. We evaluated Ki67 and MMP11 protein expression. The subcutaneous tumors of the lv-sh-LMNB1 group showed fewer Ki67- and MMP11-positive cells than those of the control group (Figure 6(b)). Ki67 is widely used to detect the cell proliferation abilities of malignancies. MMP11 has functional roles in tumor metastasis [30]. These findings reinforced the idea that LMNB1 could facilitate HCC proliferation and metastasis.

EMT is crucial for HCC metastasis, we evaluated the protein levels of EMT markers to demonstrate the role of LMNB1 in EMT. Western blot analysis showed that Slug, Snail, MMP11 and N-cadherin were significantly decreased at the protein level after LMNB1 was knocked down (Figure 6(c)).

### GSEA outlines LMNB1-related oncogenic signaling pathways

**Figure 7.**
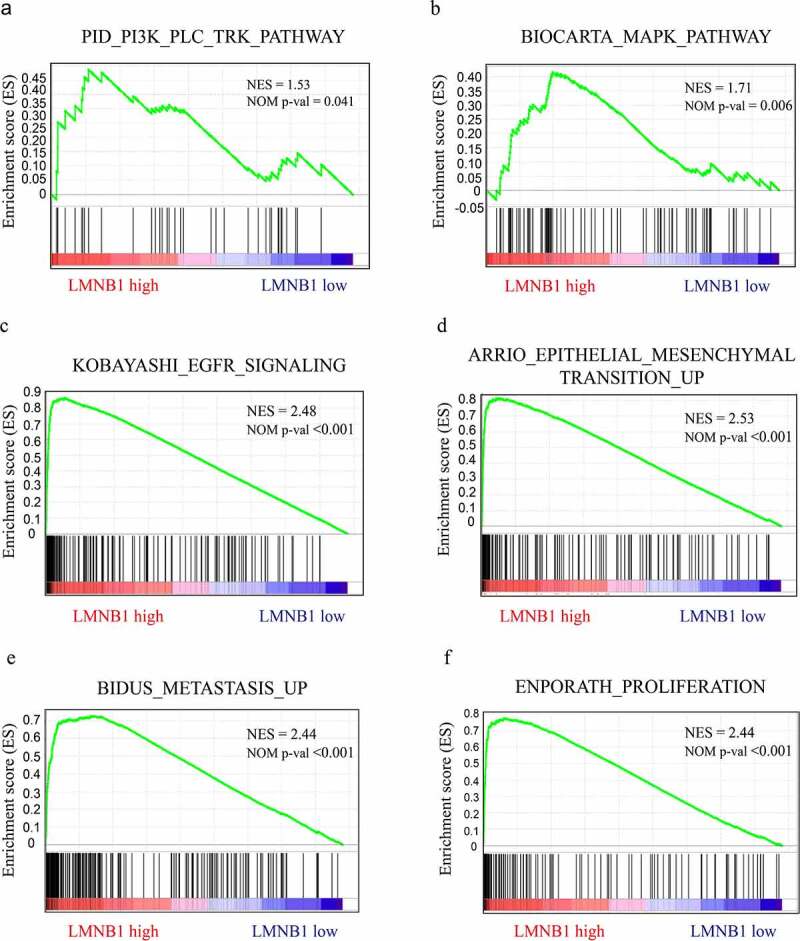
GSEA in HCC samples with LMNB1^high^ versus LMNB1^low^. (a-f) GSEA showed that increased LMNB1 expression was positively correlated with the PI3K (a), MAPK (b), and EGFR (c) pathways and had functional roles in the epithelial-mesenchymal transition (d), metastasis (e) and proliferation (f) of HCC. NES, normalized enrichment score.

GSEA based on the TCGA-LIHC dataset was performed to outline the molecular mechanisms underlying LMNB1-mediated HCC progression. As the GSEA results showed, HCC with high expression of LMNB1 was involved in the PI3K (Figure 7(a)), MAPK (Figure 7(b)) and epidermal growth factor receptor (EGFR) (Figure 7(c)) pathways. The epithelial-mesenchymal transition procedure (Figure 7(d)), BIDUS METASTASIS UP (Figure 7(e)) and BENPORATH PROLIFERATION (Figure 7(f)) gene sets showed significant enrichment in HCC with LMNB high expression.

### RNA-seq verifies that LMNB1 plays an oncogenic role through multiple pathways

**Figure 8.**
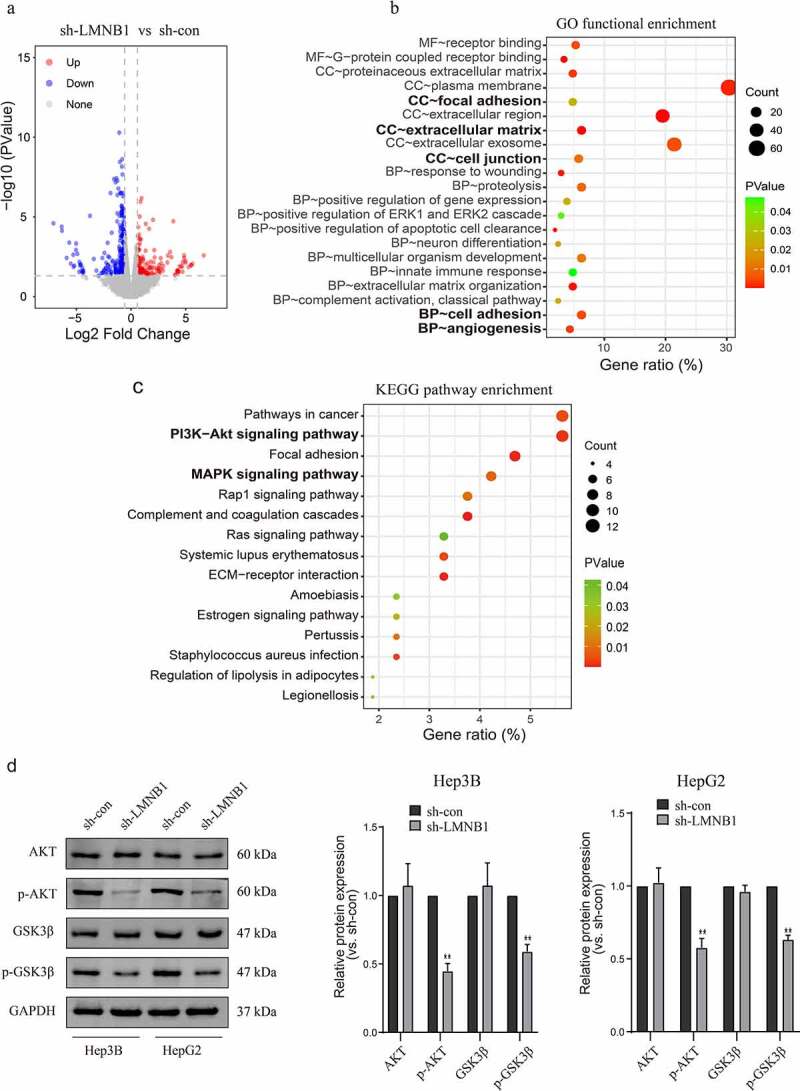
LMNB1 promotes HCC progression by regulating tumor-associated pathways. (a) Volcano plot of the DEGs. (b) GO functional enrichment analysis of the DEGs. (c) KEGG pathway enrichment analysis revealed that LMNB1 was a regulator of the PI3K and MAPK pathways in HCC. (d) Western blot analysis confirmed that p-AKT and p-GSK3β were downregulated after attenuating LMNB1 expression. *P < 0.05, **P < 0.01, ***P < 0.001.

To further verify the mechanisms by which LMNB1 promotes HCC progression, RNA-seq was carried out in LMNB1 knockdown (sh-LMNB1) HepG2 cells and control (sh-con) HepG2 cells. We screened DEGs between sh-LMNB1 HepG2 cells and sh-con HepG2 cells. The DEGs are shown in the volcano plot (Figure 8(a)). GO enrichment analysis revealed that the DEGs have functional roles in focal adhesion, the extracellular matrix, cell junctions, cell adhesion and angiogenesis (Figure 8(b)). These functions are considered to be associated with tumor metastasis. KEGG pathway enrichment analysis highlighted that LMNB1 was involved in the regulation of the PI3K-AKT and MAPK pathways (Figure 8(c)). All these pathways play a pivotal role in tumor progression. Western blot analysis showed that phosphorylated AKT and phosphorylated GSK3β were downregulated after LMNB1 was knocked down (Figure 8(d)). The above results revealed that LMNB1 promoted HCC metastasis and played an oncogenic role through multiple pathways.

### Construction and validation of a nomogram

**Figure 9.**
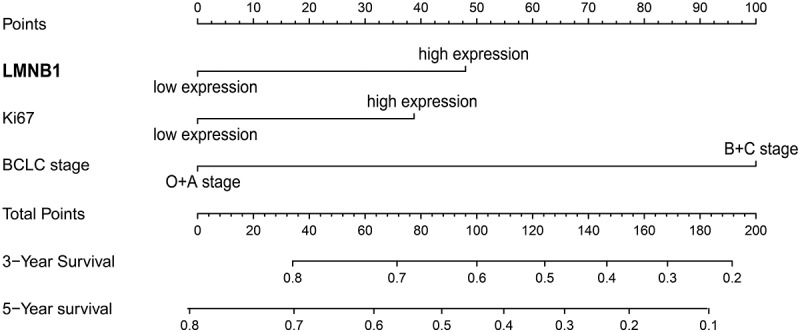
A nomogram for predicting the OS of HCC patients.

**Figure 10.**
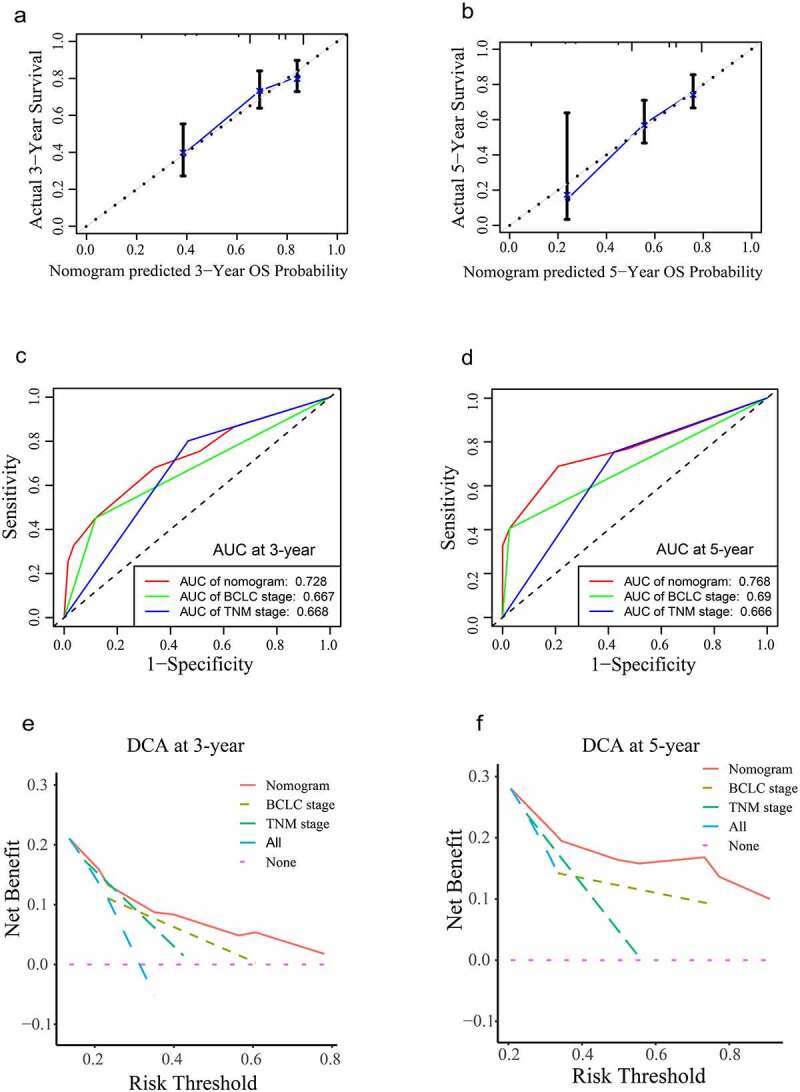
Validation of the nomogram. (a, b) Calibration curves of the nomogram for predicting OS at 3 years (a) and 5 years (b). (c, d) The accuracies of the nomogram, BCLC stage and TNM stage at 3 years (c) and 5 years (d) were evaluated by ROC analyses. (e, f) The DCA results showed that the nomogram had the highest benefit compared to the BCLC stage and TNM stage.

The Schönfeld test showed that the independent risk factors LMNB1, Ki67 and BCLC stage meet the PH assumption (all P > 0.05, Figure S1). We constructed a predictive nomogram using these independent risk factors (Figure 9). Then, we employed calibration plots and ROC curves to validate the nomogram. Calibration plots indicated that our nomogram had a favorable predictive accuracy. As shown in (Figure 10(a,b)), the predicted OS at 3 and 5 years was approximately the same as the actual OS. In the ROC curves, the area under the curves (AUCs) of our nomogram were drawn to predict OS at 3 and 5 years. The AUCs of the nomogram, BCLC stage and TNM stage for predicting 3-year OS were 0.728, 0.667 and 0.668, respectively (Figure 10(c)). The AUCs of the nomogram, BCLC stage and TNM stage for predicting 5-year OS were 0.768, 0.69 and 0.666, respectively (Figure 10(d)). The nomogram displayed better predictive accuracy than the BCLC stage and TNM stage. Finally, we assessed the clinical application of this nomogram by employing the DCA method. The nomogram showed a superior net benefit than the BCLC stage and TNM stage (Figure 10(e,f)). The above findings suggested that the nomogram based on LMNB1, Ki67 and BCLC stage exhibited excellent predictive value for HCC patients.

## Discussion

HCC is a heterogeneous disease. Patients with HCC are often diagnosed at a late stage and therefore suffer dismal prognoses. Therefore, it is urgently needed to discover efficient biomarkers for HCC therapy and prognosis prediction. Due to the high level of heterogeneity, personalized treatment strategies for patients with HCC are encouraged [31]. Combination analysis of multiple datasets is considered to be a more reliable method than single dataset analysis. In the present study, we integrated three datasets from TCGA-LIHC, GSE14520, and TIMER2 to explore hub genes of HCC. Through WGCNA, we found that the black gene module was closely related to HCC metastasis and survival status. By performing differential expression analysis, we identified DEGs that were associated with HCC metastasis. LMNB1 belonged to both the black gene module and DEGs. Hence, LMNB1 may promote HCC metastasis and could be considered an effective biomarker for predicting prognosis. However, the significance of LMNB1 and LMNB1-associated mechanisms in HCC has not been elucidated. Therefore, our study aims to comprehensively analyze the expression, correlation with clinical features, prognostic value, biological functions and potential mechanisms of LMNB1 in HCC. An additional purpose of our research is to construct a reliable predictive nomogram based on LMNB1, Ki67 and BCLC stage for HCC.

Lamins play a pivotal role in maintaining intracellular physiological equilibrium. The lamin family includes LMNA, LMNB1, LMNB2 and LMNC [32,33]. Previous studies reported that LMNB1 is essential for the survival of mammalian cells [34,35]. In addition, accumulating evidence suggests that LMNB1 is abnormally expressed in multiple malignant tumors, such as lung adenocarcinoma [36], gastric cancer [13], renal cell carcinoma [15] and B-cell malignancies [37]. The dysregulation of LMNB1 is associated with tumor progression. Luo et al. reported that overexpression of LMNB1 promotes prostate cancer metastasis [38]. In our report, we systematically evaluated the expression features of LMNB1 in HCC and peritumoral tissues. Based on the integrated analysis of the TCGA-LIHC dataset, GSE14520 dataset and TIMER2 database, we found that LMNB1 was overexpressed in HCC, especially in HCC with high metastasis risk. Enhanced protein levels of LMNB1 were also verified in cell lines and tissues from Nanfang Hospital. Moreover, enhanced LMNB1 expression was found to be closely correlated with advanced TNM stage, poorer histological differentiation, higher levels of AFP, tumor recurrence and tumor metastasis. Our results also revealed that LMNB1 was independently correlated with overall survival. These results indicate that LMNB1 is not only related to the occurrence of HCC but also may be involved in its development, including tumor recurrence and metastasis. Notably, these results are highly consistent with previous studies. For example, Sun et al. reported that overexpression of LMNB1 was significantly associated with advanced TNM stage [16]. Tang et al. identified that LMNB1 knockdown reduced cell migration ability in lung adenocarcinoma. All these findings demonstrated that LMNB1 may act as a tumor promoter and prognostic marker for HCC.

Through in vitro loss-of-function assays, we further verified the biological functions of LMNB1 in HCC. The results showed that LMNB1 knockdown markedly attenuated the proliferation and metastasis abilities of HCC. Moreover, LMNB1 knockdown inhibited the EMT process in vitro. These results were consistent with our bioinformatic analysis. Cancer metastasis leads to over 90% of human cancer-related deaths [39]. Inhibiting the metastasis process provides great benefit for the treatment of cancer patients [40]. Yang et al. reported that Huaier could inhibit prostate cancer proliferation and metastasis by suppressing the expression of LMNB1 [41]. All these findings suggested that inhibitors targeting LMNB1 may contribute to the treatment of HCC.

We conducted GSEA on the TCGA-LIHC dataset and performed functional annotation and enrichment pathway analyses on our RNA-seq data to better understand the LMNB1-associated mechanisms in HCC. The pathway analysis results indicated that LMNB1-related signaling pathways in HCC were enriched in the PI3K, MAPK and EGFR pathways. PI3K signaling is an important intracellular pathway [42]. PI3K pathway activation is frequently observed in various tumors and leads to poor survival. A recent study reported that CD73 facilitates the development of HCC by regulating the PI3K/AKT pathway [43]. Our experiments showed that phosphorylated AKT and phosphorylated GSK3β were downregulated after LMNB1 knockout, confirming that LMNB1 positively regulated PI3K signaling. MAPK and EGFR signaling are other important pathways that promote the malignant behavior of HCC. Phosphorylation of MAPK signaling regulates the proliferation, differentiation, apoptosis and angiogenesis of HCC [44]. Sueangoen and Wang et al. reported that EGFR is upregulated in various human cancers, and high expression of EGFR leads to dismal clinical outcomes [45,46]. Moreover, the results of functional annotation analysis were concentrated on tumor metastasis-related biological processes, including epithelial-mesenchymal transition, focal adhesion, extracellular matrix, cell junctions and cell adhesion, reinforcing the idea that LMNB1 promotes HCC metastasis. The above findings revealed that LMNB1 could promote HCC progression by regulating tumor-associated pathways. Thus, suppression of LMNB1 and LMNB1-related signaling pathways may be a novel strategy to treat HCC. However, more experimental verifications are needed to validate our findings.

Accurate prognostic assessment is pivotal for choosing an appropriate treatment protocol. The BCLC stage and TNM stage are widely used for treatment strategy establishment and clinical outcome prediction in HCC. However, various clinical outcomes are observed among HCC patients with the same BCLC stage or TNM stage [21,47]. The BCLC staging system focuses on tumor status and clinical information. The TNM staging system is based on tumor anatomical information. HCC is a heterogeneous disease [48]. Genomic heterogeneity is not taken into consideration may limit the predictive abilities of these systems. Biomarkers have potential prognostic value for HCC. Feng et al. found that the expression of ARID1A could be used to predict the outcome of HCC [49]. Moreover, previous studies have integrated several molecular biomarkers into HCC prognostic models [50,51]. Surprisingly, the prognostic accuracy of these gene signatures is equal to or even superior to the TNM stage. In this study, we incorporated LMNB1, Ki67 and BCLC stage into a nomogram. Ki67 is a well-known proliferation biomarker. Surprisingly, the points contributed by high LMNB1 expression were higher than the points contributed by high Ki67 expression. Another encouraging finding is that the ROC analysis and DCA result demonstrated that our nomogram performed better than the TNM stage and BCLC stage, which suggests that LMNB1 could add prognostic value to the BCLC stage and contribute to individualized treatment for patients with HCC.

## Conclusion

In summary, we showed that LMNB1 is overexpressed in HCC through combined database analysis and experimental validation. High LMNB1 expression is correlated with aggressive clinicopathological features and dismal survival. LMNB1 could promote HCC proliferation and metastasis and regulate the PI3K and MAPK pathways. In addition, incorporating LMNB1, Ki67 and BCLC stage into a nomogram achieved strong survival predictive accuracy. Thus, LMNB1 may serve as an effective therapeutic target and a reliable prognostic biomarker for HCC.

## Supplementary Material

Supplemental Material

## Data Availability

The public data included in this study originate from The Gene Expression Omnibus (https://www.ncbi.nlm.nih.gov/geo/), The Cancer Genome Atlas (https://portal.gdc.cancer.gov/) and the TIMER2 database (http://timer.cistrome.org/). The experimental data that support the findings of this study are available on request from the corresponding author.

## Highlights

- LMNB1 is overexpressed in HCC.
- LMNB1 expression is significantly correlated with aggressive clinicopathological features. Overexpression of LMNB1 indicates a dismal prognosis of HCC.
- LMNB1 regulates epithelial-mesenchymal transition (EMT) and tumorigenesis via the PI3K/MAPK pathway in HCC.
- LMNB1 is an effective therapeutic target for HCC.
- The nomogram may contribute to individualized treatment for patients with HCC.

## References

[1] Sung H, Ferlay J, Siegel RL, et al. Global cancer Statistics 2020: GLOBOCAN estimates of incidence and mortality worldwide for 36 cancers in 185 countries. CA Cancer J Clin. 2021;71(3):209–249. 33538338 10.3322/caac.21660

[2] Raoul JL, Forner A, Bolondi L, et al. Updated use of TACE for hepatocellular carcinoma treatment: how and when to use it based on clinical evidence. Cancer Treat Rev. 2019;72:28–36. 30447470 10.1016/j.ctrv.2018.11.002

[3] Chen L, Sun J, Yang X. Radiofrequency ablation-combined multimodel therapies for hepatocellular carcinoma: current status. Cancer Lett. 2016;370(1):78–84. 26472630 10.1016/j.canlet.2015.09.020 PMC4686130

[4] Chidambaranathan-Reghupaty S, Fisher PB, Sarkar D. Hepatocellular carcinoma (HCC): epidemiology, etiology and molecular classification. Adv Cancer Res. 2021;149:1–61. 33579421 10.1016/bs.acr.2020.10.001 PMC8796122

[5] Ghavimi S, Apfel T, Azimi H, et al. Management and treatment of hepatocellular carcinoma with immunotherapy: a review of current and future options. J Clin Transl Hepatol. 2020;8(2):168–176. 32832397 10.14218/JCTH.2020.00001 PMC7438354

[6] Chen S, Cao Q, Wen W, et al. Targeted therapy for hepatocellular carcinoma: challenges and opportunities. Cancer Lett. 2019;460:1–9. 31207320 10.1016/j.canlet.2019.114428

[7] Tang W, Chen Z, Zhang W, et al. The mechanisms of sorafenib resistance in hepatocellular carcinoma: theoretical basis and therapeutic aspects. Signal Transduct Target Ther. 2020;5(1):87. 32532960 10.1038/s41392-020-0187-x PMC7292831

[8] Camps J, Erdos MR, Ried T. The role of lamin B1 for the maintenance of nuclear structure and function. Nucleus. 2015;6(1):8–14. 25602590 10.1080/19491034.2014.1003510 PMC4615282

[9] Hutchison CJ. B-type lamins in health and disease. Semin Cell Dev Biol. 2014;29:158–163. 24380701 10.1016/j.semcdb.2013.12.012 PMC4053831

[10] Dechat T, Pfleghaar K, Sengupta K, et al. Nuclear lamins: major factors in the structural organization and function of the nucleus and chromatin. Genes Dev. 2008;22(7):832–853. 18381888 10.1101/gad.1652708 PMC2732390

[11] Brady GF, Kwan R, Bragazzi Cunha J, et al. Lamins and lamin-associated proteins in gastrointestinal health and disease. Gastroenterology. 2018;154(6):1602–1619. 29549040 10.1053/j.gastro.2018.03.026 PMC6038707

[12] Izdebska M, Gagat M, Grzanka A. Overexpression of lamin B1 induces mitotic catastrophe in colon cancer LoVo cells and is associated with worse clinical outcomes. Int J Oncol. 2017;52(1):89–102. 29115590 10.3892/ijo.2017.4182 PMC5743383

[13] Yu ZY, Jiang XY, Zhao RR, et al. Lamin B1 deficiency promotes malignancy and predicts poor prognosis in gastric cancer. Neoplasma. 2020;67(6):1303. 32787434 10.4149/neo_2020_200109N33

[14] Li L, Du Y, Kong X, et al. Lamin B1 is a novel therapeutic target of betulinic acid in pancreatic cancer. Clin Cancer Res. 2013;19(17):4651–4661. 23857605 10.1158/1078-0432.CCR-12-3630 PMC3800003

[15] Radspieler MM, Schindeldecker M, Stenzel P, et al. Lamin-B1 is a senescence-associated biomarker in clear-cell renal cell carcinoma. Oncol Lett. 2019;18(3):2654–2660. 31402955 10.3892/ol.2019.10593 PMC6676677

[16] Sun S, Xu MZ, Poon RT, et al. Circulating Lamin B1 (LMNB1) biomarker detects early stages of liver cancer in patients. J Proteome Res. 2010;9(1):70–78. 19522540 10.1021/pr9002118

[17] Wong KF, Luk JM. Discovery of lamin B1 and vimentin as circulating biomarkers for early hepatocellular carcinoma. Methods Mol Biol. 2012;909:295–310. 22903723 10.1007/978-1-61779-959-4_19

[18] Tellapuri S, Sutphin PD, Beg MS, et al. Staging systems of hepatocellular carcinoma: a review. Indian J Gastroenterol. 2018;37(6):481–491. 30593649 10.1007/s12664-018-0915-0

[19] Burkhart RA, Pawlik TM. Staging and prognostic models for hepatocellular carcinoma and intrahepatic cholangiocarcinoma. Cancer Control. 2017;24(3):544013179. 10.1177/1073274817729235 PMC5937249 28975828

[20] Giannini EG, Bucci L, Garuti F, et al. Patients with advanced hepatocellular carcinoma need a personalized management: a lesson from clinical practice. Hepatology. 2018;67(5):1784–1796. 29159910 10.1002/hep.29668

[21] Golfieri R, Bargellini I, Spreafico C, et al. Patients with Barcelona clinic liver cancer stages b and c hepatocellular carcinoma: time for a subclassification. Liver Cancer. 2019;8(2):78–91. 31019899 10.1159/000489791 PMC6465743

[22] Zhou W, Zhang Y, Zhang S, et al. Absent in melanoma 1-like (AIM1L) serves as a novel candidate for overall survival in hepatocellular carcinoma. Bioengineered. 2021;12(1):2750–2762. 34130591 10.1080/21655979.2021.1939636 PMC8806546

[23] Ouyang G, Yi B, Pan G, et al. A robust twelve-gene signature for prognosis prediction of hepatocellular carcinoma. Cancer Cell Int. 2020;20:207. 32514252 10.1186/s12935-020-01294-9 PMC7268417

[24] Long J, Zhang L, Wan X, et al. A four‐gene‐based prognostic model predicts overall survival in patients with hepatocellular carcinoma. J Cell Mol Med. 2018;22(12):5928–5938. 30247807 10.1111/jcmm.13863 PMC6237588

[25] Langfelder P, Horvath S. WGCNA: an R package for weighted correlation network analysis. Bmc Bioinformatics. 2008;9(1):559. 19114008 10.1186/1471-2105-9-559 PMC2631488

[26] Hanzelmann S, Castelo R, Guinney J. GSVA: gene set variation analysis for microarray and RNA-seq data. Bmc Bioinformatics. 2013;14:7. 23323831 10.1186/1471-2105-14-7 PMC3618321

[27] Ramos-Vara JA. Principles and Methods of Immunohistochemistry. Methods Mol Biol. 2017;1641:115–128. 28748460 10.1007/978-1-4939-7172-5_5

[28] Huang Y, Xiang B, Liu Y, et al. LncRNA CDKN2B-AS1 promotes tumor growth and metastasis of human hepatocellular carcinoma by targeting let-7c-5p/NAP1L1 axis. Cancer Lett. 2018;437:56–66. 30165194 10.1016/j.canlet.2018.08.024

[29] Hung JH, Yang TH, Hu Z, et al. Gene set enrichment analysis: performance evaluation and usage guidelines. Brief Bioinform. 2012;13(3):281–291. 21900207 10.1093/bib/bbr049 PMC3357488

[30] Kou YB, Zhang SY, Zhao BL, et al. Knockdown of MMP11 inhibits proliferation and invasion of gastric cancer cells. Int J Immunopathol Pharmacol. 2013;26(2):361–370. 23755751 10.1177/039463201302600209

[31] Bruix J, Han K, Gores G, et al. Liver cancer: approaching a personalized care. J Hepatol. 2015;62(1):S144–S156. 25920083 10.1016/j.jhep.2015.02.007 PMC4520430

[32] Lin F, Worman HJ. Structural organization of the human gene encoding nuclear lamin A and nuclear lamin C. J Biol Chem. 1993;268(22):16321–16326. 8344919

[33] Lin F, Worman HJ. Structural organization of the human gene (LMNB1) encoding nuclear lamin B1. Genomics. 1995;27(2):230–236. 7557986 10.1006/geno.1995.1036

[34] Yang SH, Jung H, Coffinier C, et al. Are B-type lamins essential in all mammalian cells? Nucleus. 2011;2(6):562–569. 22127257 10.4161/nucl.2.6.18085 PMC3324344

[35] Harborth J, Elbashir SM, Bechert K, et al. Identification of essential genes in cultured mammalian cells using small interfering RNAs. J Cell Sci. 2001;114(Pt 24):4557–4565. 11792820 10.1242/jcs.114.24.4557

[36] Jia Y, Vong JS, Asafova A, et al. Lamin B1 loss promotes lung cancer development and metastasis by epigenetic derepression of RET. J Exp Med. 2019;216(6):1377–1395. 31015297 10.1084/jem.20181394 PMC6547854

[37] Klymenko T, Bloehdorn J, Bahlo J, et al. Lamin B1 regulates somatic mutations and progression of B-cell malignancies. Leukemia. 2018;32(2):364–375. 28804121 10.1038/leu.2017.255 PMC5808072

[38] Luo F, Han J, Chen Y, et al. Lamin B1 promotes tumor progression and metastasis in primary prostate cancer patients. Future Oncol. 2021;17(6):663–673. 33112662 10.2217/fon-2020-0825

[39] Leverrier-Penna S, Destaing O, Penna A. Insights and perspectives on calcium channel functions in the cockpit of cancerous space invaders. Cell Calcium. 2020;90:102251. 32683175 10.1016/j.ceca.2020.102251

[40] Yang L, Qiu J, Xiao Y, et al. AP-2beta inhibits hepatocellular carcinoma invasion and metastasis through Slug and Snail to suppress epithelial-mesenchymal transition. Theranostics. 2018;8(13):3707–3721. 30026878 10.7150/thno.25166 PMC6037033

[41] Yang A, Zhao Y, Wang Y, et al. Huaier suppresses proliferative and metastatic potential of prostate cancer PC3 cells via downregulation of Lamin B1 and induction of autophagy. Oncol Rep. 2018;39(6):3055–3063. 29658595 10.3892/or.2018.6358

[42] Yang J, Nie J, Ma X, et al. Targeting PI3K in cancer: mechanisms and advances in clinical trials. Mol Cancer. 2019;18(1):26. 30782187 10.1186/s12943-019-0954-x PMC6379961

[43] Ma X, Shen M, Hu B, et al. CD73 promotes hepatocellular carcinoma progression and metastasis via activating PI3K/AKT signaling by inducing Rap1-mediated membrane localization of P110β and predicts poor prognosis. J Hematol Oncol. 2019;12(1): 37. 10.1186/s13045-019-0724-7 PMC6458749 30971294

[44] Diniz PHC, Silva SDC, Vidigal PVT, et al. Expression of MAPK and PI3K/AKT/mTOR proteins according to the chronic liver disease etiology in hepatocellular carcinoma. J Oncol. 2020;2020:1–9. 10.1155/2020/4609360 PMC7644337 33178273

[45] Sueangoen N, Tantiwetrueangdet A, Panvichian R. HCC-derived EGFR mutants are functioning, EGF-dependent, and erlotinib-resistant. Cell Biosci. 2020;10(1):41. 10.1186/s13578-020-00407-1 PMC7076995 32190291

[46] Wang W, Ma X, Shi Z, et al. Epidermal growth factor receptor pathway polymorphisms and the prognosis of hepatocellular carcinoma. Am J Cancer Res. 2015;5(1):396–410. 25628948 PMC4300692

[47] Pawlik TM, Esnaola NF, Vauthey JN. Surgical treatment of hepatocellular carcinoma: similar long-term results despite geographic variations. Liver Transpl. 2004;10(2 Suppl 1):S74–S80. 10.1002/lt.20052 14762844

[48] Li L, Wang H. Heterogeneity of liver cancer and personalized therapy. Cancer Lett. 2016;379(2):191–197. 26213370 10.1016/j.canlet.2015.07.018

[49] Feng Y, Tang X, Li C, et al. ARID1A is a prognostic biomarker and associated with immune infiltrates in hepatocellular carcinoma. Can J Gastroenterol Hepatol. 2022;2022:3163955. 35028302 10.1155/2022/3163955 PMC8752298

[50] Yang Z, Zi Q, Xu K, et al. Development of a macrophages-related 4-gene signature and nomogram for the overall survival prediction of hepatocellular carcinoma based on WGCNA and LASSO algorithm. Int Immunopharmacol. 2021;90:107238. 33316739 10.1016/j.intimp.2020.107238

[51] Dai Y, Qiang W, Lin K, et al. An immune-related gene signature for predicting survival and immunotherapy efficacy in hepatocellular carcinoma. Cancer Immunol Immunother. 2021;70(4):967–979. 33089373 10.1007/s00262-020-02743-0 PMC10992402

