# Supplementary material for: Lamin B1 is a potential therapeutic target and prognostic biomarker for hepatocellular carcinoma
Source: Bioengineered. 2022 Apr 18;13(4):9211–31. doi: 10.1080/21655979.2022.2057896 (PMC9161935; doi:10.1080/21655979.2022.2057896)
Supplement: Supplemental Material [file KBIE_A_2057896_SM1762.zip › Supplementary materials/Supplementary Table 2.docx]

Supplementary Table 2. Specific information of HCC patients (n=20)

| Characteristics | No. of cases (%) |
| --- | --- |
| Age (years) |  |
| <60 | 11 (55) |
| ≥60 | 9 (45) |
| Gender |  |
| Male | 14 (70) |
| Female | 6 (30) |
| BMI |  |
| <25 | 12 (60) |
| ≥25 | 8 (40) |
| AFP level |  |
| <300 | 13 (65) |
| ≥300 | 7 (35) |
| Grade |  |
| G1 | 2 (10) |
| G2 | 14 (70) |
| G3 | 4 (30) |
| TNM stage |  |
| I | 4 (20) |
| II | 8 (40) |
| III | 8 (40) |
| Vascular invasion |  |
| Yes | 8 (40) |
| No | 12 (40) |
