# Supplementary material for: Lamin B1 is a potential therapeutic target and prognostic biomarker for hepatocellular carcinoma
Source: Bioengineered. 2022 Apr 18;13(4):9211–31. doi: 10.1080/21655979.2022.2057896 (PMC9161935; doi:10.1080/21655979.2022.2057896)
Supplement: Supplemental Material [file KBIE_A_2057896_SM1762.zip › Supplementary materials/Supplementary Table 6.docx]

Supplementary Table 6. Correlation between LMNB1 expression and clinicopathological characteristics of HCC patients from TCGA-LIHC dataset

| **Characteristics** | **N** | **LMNB1^low^** | **LMNB1^high^** | **P value** |
| --- | --- | --- | --- | --- |
| Age (years)  <60  ≥60  NA | 167  196  1 | 71  111 | 96  85 | 0.007 |
| Gender  Female  Male | 118  246 | 54  128 | 64  118 | 0.263 |
| BMI  ≤24  >24  NA | 158  173  33 | 71  96 | 87  77 | 0.055 |
| AFP level  ≤400  >400  NA | 209  65  90 | 126  15 | 83  50 | <0.001 |
| Grade  G1+ G2  G3+ G4  NA | 228  132  4 | 134  46 | 94  86 | <0.001 |
| Tumor status  Tumor free  With tumor  NA | 159  119  86 | 92  50 | 67  69 | 0.009 |
| Metastatic score  Low (Cluster1)  High(Cluster2) | 196  168 | 160  22 | 36  146 | <0.001 |
| TNM stage  I  II  III+ IV  NA | 167  84  89  24 | 97  36  36 | 70  48  53 | 0.009 |
