# Supplementary figures and images for: Lamin B1 is a potential therapeutic target and prognostic biomarker for hepatocellular carcinoma
Source: Bioengineered. 2022 Apr 18;13(4):9211–31. doi: 10.1080/21655979.2022.2057896 (PMC9161935; doi:10.1080/21655979.2022.2057896)

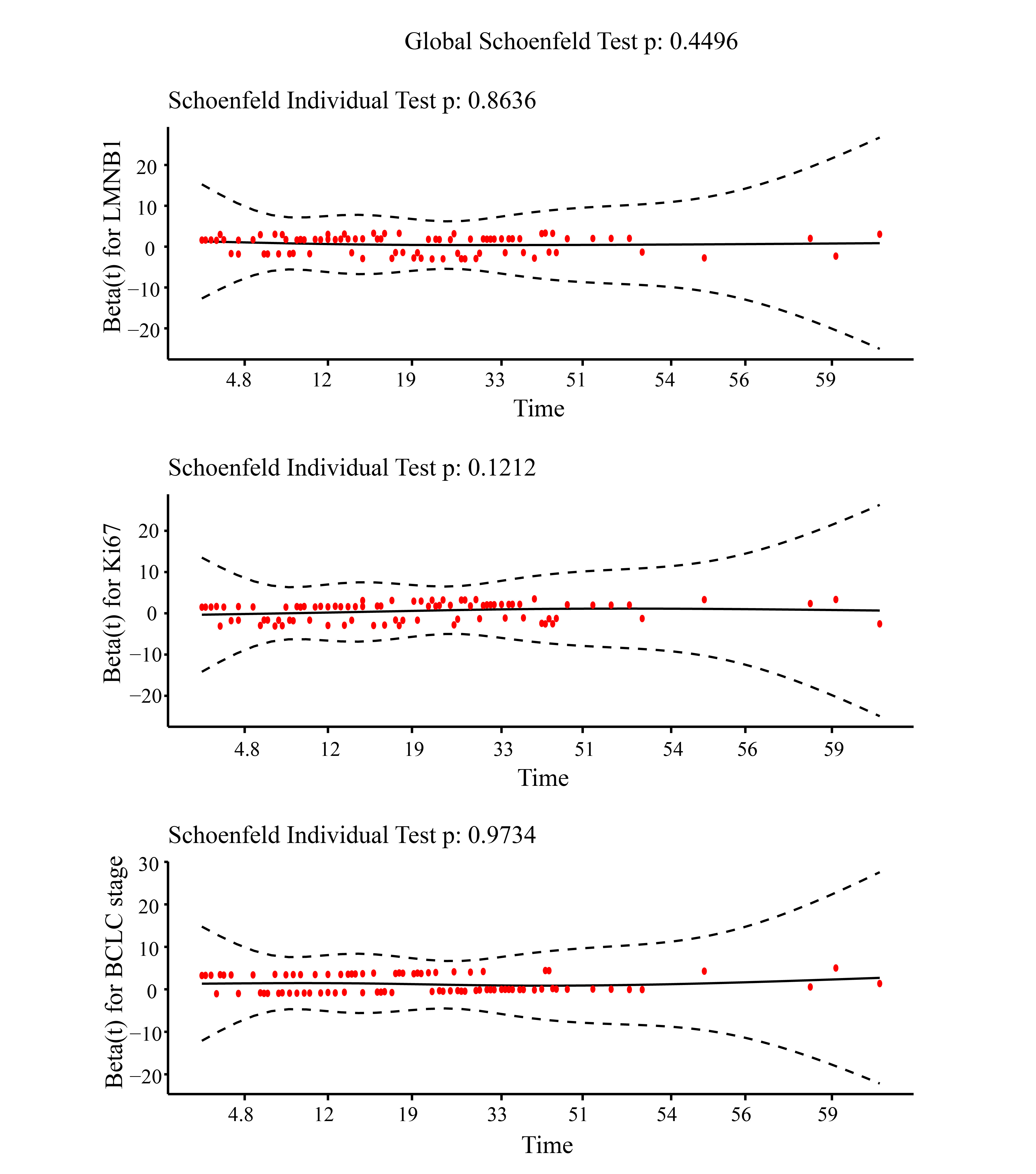


Figure S1. Schönfeld test of PH assumption. P >0.05 indicates the variable meets the PH assumption.

Supplement: Supplemental Material [file KBIE_A_2057896_SM1762.zip › Supplementary materials/Figure S1.docx]
